# Development of an Immune-Pathology Informed Radiomics Model for Non-Small Cell Lung Cancer

**DOI:** 10.1038/s41598-018-20471-5

**Published:** 2018-01-31

**Authors:** Chad Tang, Brian Hobbs, Ahmed Amer, Xiao Li, Carmen Behrens, Jaime Rodriguez Canales, Edwin Parra Cuentas, Pamela Villalobos, David Fried, Joe Y. Chang, David S. Hong, James W. Welsh, Boris Sepesi, Laurence Court, Ignacio I. Wistuba, Eugene J. Koay

**Affiliations:** 1 Department of Radiation Oncology, The University of Texas MD Anderson Cancer Center, Houston, TX USA; 2 Department of Biostatistics, The University of Texas MD Anderson Cancer Center, Houston, TX USA; 3 Department of Thoracic Head & Neck Medical Oncology, The University of Texas MD Anderson Cancer Center, Houston, TX USA; 4 Department of Translational Molecular Pathology, The University of Texas MD Anderson Cancer Center, Houston, TX USA; 5 Department of Investigational Cancer Therapeutics, The University of Texas MD Anderson Cancer Center, Houston, TX USA; 6 Department of Surgery, The University of Texas MD Anderson Cancer Center, Houston, TX USA; 7 Department of Radiation Physics, The University of Texas MD Anderson Cancer Center, Houston, TX USA

## Abstract

With increasing use of immunotherapy agents, pretreatment strategies for identifying responders and non-responders is useful for appropriate treatment assignment. We hypothesize that the local immune micro-environment of NSCLC is associated with patient outcomes and that these local immune features exhibit distinct radiologic characteristics discernible by quantitative imaging metrics. We assembled two cohorts of NSCLC patients treated with definitive surgical resection and extracted quantitative parameters from pretreatment CT imaging. The excised primary tumors were then quantified for percent tumor PDL1 expression and density of tumor-infiltrating lymphocyte (via CD3 count) utilizing immunohistochemistry and automated cell counting. Associating these pretreatment radiomics parameters with tumor immune parameters, we developed an immune pathology-informed model (IPIM) that separated patients into 4 clusters (designated A-D) utilizing 4 radiomics features. The IPIM designation was significantly associated with overall survival in both training (5 year OS: 61%, 41%, 50%, and 91%, for clusters A-D, respectively, P = 0.04) and validation (5 year OS: 55%, 72%, 75%, and 86%, for clusters A-D, respectively, P = 0.002) cohorts and immune pathology (all P < 0.05). Specifically, we identified a favorable outcome group characterized by low CT intensity and high heterogeneity that exhibited low PDL1 and high CD3 infiltration, suggestive of a favorable immune activated state. We have developed a NSCLC radiomics signature based on the immune micro-environment and patient outcomes. This manuscript demonstrates model creation and validation in independent cohorts.

## Introduction

Immunotherapy has shown promise for the treatment of several types of cancer, including non-small cell lung cancer (NSCLC)^1,2^, which nevertheless remains the leading cause of cancer death in the United States^3,4^. Indeed, anti-PD1 and anti-CTLA4 checkpoint inhibitors have led to responses in a proportion of NSCLC patients, but many patients do not respond^5–8^. Efforts are underway to identify predictors of response, yet most such efforts are limited to analyses of biopsy-based pathologic biomarkers or serum samples^9,10^. One rich source of biophysical data that has not been thoroughly investigated for this purpose is radiomics, defined as the quantification of imaging features from CT or other imaging modalities^11–13^. As a predictive marker, radiomics has several advantages over tissue-based biomarkers, including being noninvasive, easily used for serial monitoring, implemented clinically by using standard-of-care imaging, and acquiring data from the entire tumor.

Associations between radiomics and patient outcomes have been shown for NSCLC^14–16^. However, several shortcomings have impeded the clinical utilization of radiomics, including the lack of a clear underlying biological rationale to explain the association between radiomics and outcomes in addition to the abstract mathematical nature of radiomics parameters^15,17^. Efforts thus far have identified associations between EGFR and KRAS mutations with radiomics features in NSCLC^18,19^. One of the most significant drivers of the local tumor environment, and correspondingly of patient outcomes in NSCLC, is the local inflammatory reaction^20,21^. Immune-related phenomena such as increased vascular permeability, cytokine release, lymphocyte infiltration, and fibrosis produce corresponding imaging correlates on CT^22–24^. Given emerging evidence suggesting that NSCLC is driven in substantial part by the local immune environment^7,20,21^, we hypothesized that patterns of enhancement, texture, shape, and morphology intrinsic to the local immune phenotype could be ascertained through interrogation of radiomics features. This retrospective study was devised to identify and validate image-derived prognostic signatures for NSCLC that reflect the local immune microenvironment and are prognostic for patient survival.

## Results

### Patients

Demographics for the training cohort (n = 114) and validation cohort (n = 176) are presented in Table 1. Patients in the training cohort had been treated for NSCLC from December 2000 through February 2012, and those in the validation cohort had been treated from January 2006 to December 2009. Both datasets were relatively well balanced in terms of sex (52–53% men and 47–48% women), performance status (KPS 70–100 in 94–98%), predominance of N0 disease (69–74%), and smoking status (46–53% were former smokers). The validation dataset included more patients with early T disease (T1: 48% vs 30% in the training set, *P* = 0.005), adenocarcinoma/not otherwise specific (NOS) histology (68% vs 55%, *P* = 0.007), and less common use of adjuvant therapy (26% vs 43%, *P* = 0.001).

**Table 1:** Baseline patient characteristics.

**Characteristics**	**Training Set (n = 114)**	**Validation Set (n = 176)**	**P-value (Fisher test)**
Sex			
Male	59 (52%)	94 (53%)	0.81
Female	55 (48%)	82 (47%)	
Age at Treatment, yr, median (min-max)	67 (42–84)	68 (39–87)	0.21*
Karnofsky performance status			0.11
70–100	107 (94%)	173 (98%)	
<70	7 (6%)	4 (2%)	
Tumor Histology			0.04
Adenocarcinoma/NOS	63 (55%)	119 (68%)	
Squamous	51 (45%)	57 (32%)	
T Status			0.005
T1	34 (30%)	84 (48%)	
T2	55 (48%)	71 (40%)	
T3/4	25 (22%)	21 (12%)	
N Status			0.35
N0	79 (69%)	131 (74%)	
N1/2	35 (31%)	45 (26%)	
Smoking Status			0.003
Never	5 (4%)	24 (14%)	
Former	52 (46%)	93 (53%)	
Current	57 (50%)	59 (34%)	
Adjuvant Therapy			0.002
Yes	49 (43%)	45 (26%)	
No	64 (57%)	131 (74%)	
Margin Status			
Close(<2 mm)/Positive	7 (6%)	9 (5%)	0.79
Negative	107 (94%)	167 (95%)	

Abbreviations: NOS, not otherwise specified.

*T-test utilized.

### Association of immune-pathology features with survival

**Figure 1:**
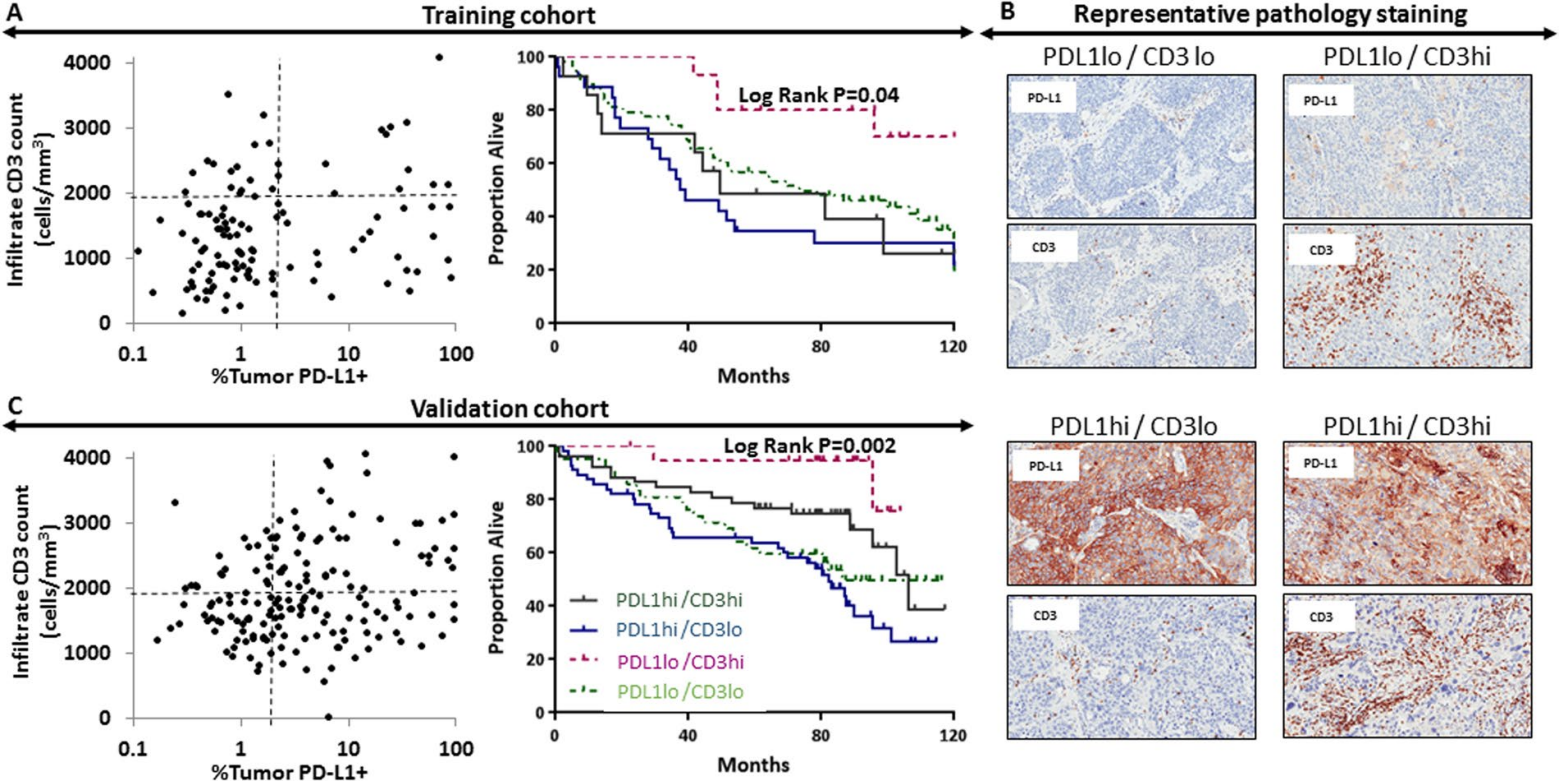
Pathology grouping based on CD3+ infiltrating lymphocyte count and PD-L1+ tumor cell count in resected tumors showing pathology-based stratification and corresponding survival in the training cohort (**A**). Representative images showing immunohistochemistry PD-L1 and CD3 staining from patients within the training cohort (**B**). Pathology groupings and survival stratification are reproduced in the validation cohort (**C**).

Resected tumor samples were stained for two pathology markers and the following metrics extracted: CD3 count (to assess lymphocyte infiltration) and % of tumor cells expressing PDL1 (to assess tumor-mediated immune suppression). In the training cohort, optimal cutoffs for both metrics were determined to maximize association with OS when partitioning this cohort into 4 immune pathology groups: PDL1 cutoff of 2% and a CD3 count cutoff of 1910 cells/mm^2^ (Fig. 1A). Representative IHC stains for CD3 and PDL1 from the 4 immune-pathology groups are presented in Fig. 1B. When considered separately, stratification by PDL1 or CD3 utilizing the identified cutpoints exhibited a trend towards a significant association with survival (P = 0.08 and P = 0.06, respectively). However, when considered together, pathology classification using both PDL1 and CD3 exhibited a significant association with survival; specifically, patients with immune-activated tumors (CD3^hi^PDL1^lo^) had the highest OS rate at 5 yr (80%), and patients with immune-inhibited tumors (CD3^lo^PDL1^hi^) had the worst OS rate at 5 yr (35%) (*P* = 0.04) (Fig. 1A). This association held on multivariate analysis considering baseline tumor and treatment factors (Supplemental Table S1).

Patients in the validation group were similarly stratified based on tumor CD3 and PDL1 staining (Fig. 1B,C). When considered separately, stratification by CD3 count exhibited a significant association with survival (P < 0.01), while stratification by PDL1 did not (P = 0.17). When considering both CD3 and PDL1 together, a similar association was noted between immune-pathology characteristics and survival; specifically, patients with immune-activated tumors had the highest 5-yr OS rate (95%), and the patients with immune-inhibited tumors had the lowest (54%) (*P* = 0.002) (Fig. 1D). This association held on multivariate analysis (Supplemental Table S1). Concordance indices based on the immune-pathology subtypes alone were 0.60 for the training set and 0.63 for the validation set.

### Creating the radiomics models

**Figure 2:**
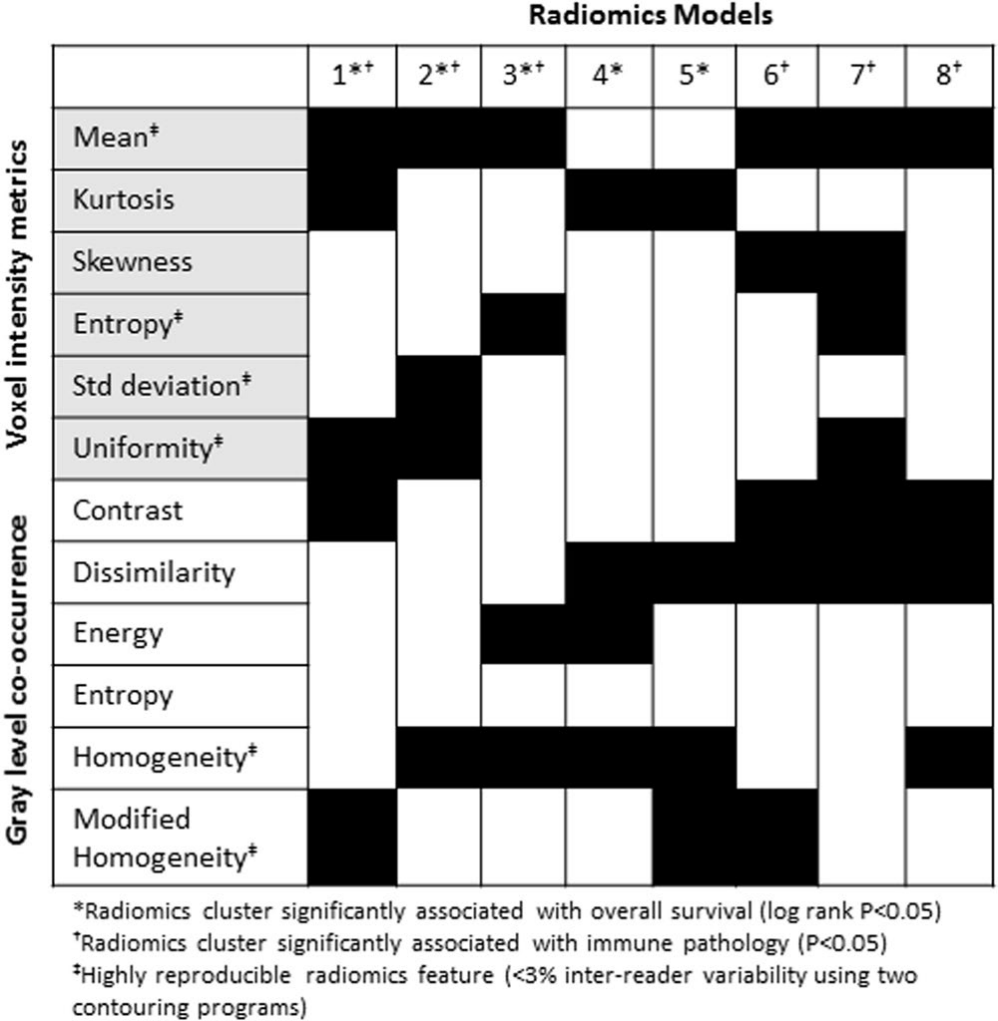
Generation of 8 distinct radiomics models, each indicated by a column. Each model utilizes a subgroup of 4–5 radiomics parameter from the 12 considered radiomics features. When considering radiomics model, (*) indicates models that exhibit a significant association with overall survival (models 1–5) and (†) indicates models that exhibit a significant association with immune pathology (models 1–3 and 6–8). When considering radiomics feature, (‡) indicates features that are highly reproducible with <3% inter-reader variability (voxel intensity mean, entropy, standard deviation, and uniformity and gray level co-occurrence homogeneity and modified homogeneity).

Hierarchical clustering of 12 prespecified radiomics features (see Methods) generated 8 independent radiomics models that were associated with immune-pathology features and/or OS (Fig. 2). Each model was produced by organizing all patients within the training dataset into radiomics clusters based on 4–5 radiomics parameters, which are a subset of the considered 12 parameters. For example, column 1 refers to radiomics model #1 which groups patients into clusters utilizing the voxel intensity metrics mean, kurtosis, and uniformity in addition to the gray level co-occurrence metrics of contrast and modified homogeneity. Of these 8 models, 3 were associated with both OS (*P* < 0.05) and immune-pathology grouping (*P* < 0.05), 1 of which was entirely composed of highly reproducible features (Supplementary Fig. S1). This model, which was associated with survival, immune pathology, and composed of highly reproducible features, is named the immune-pathology informed model (IPIM), was used for the remainder of the analyses.

**Figure 3:**
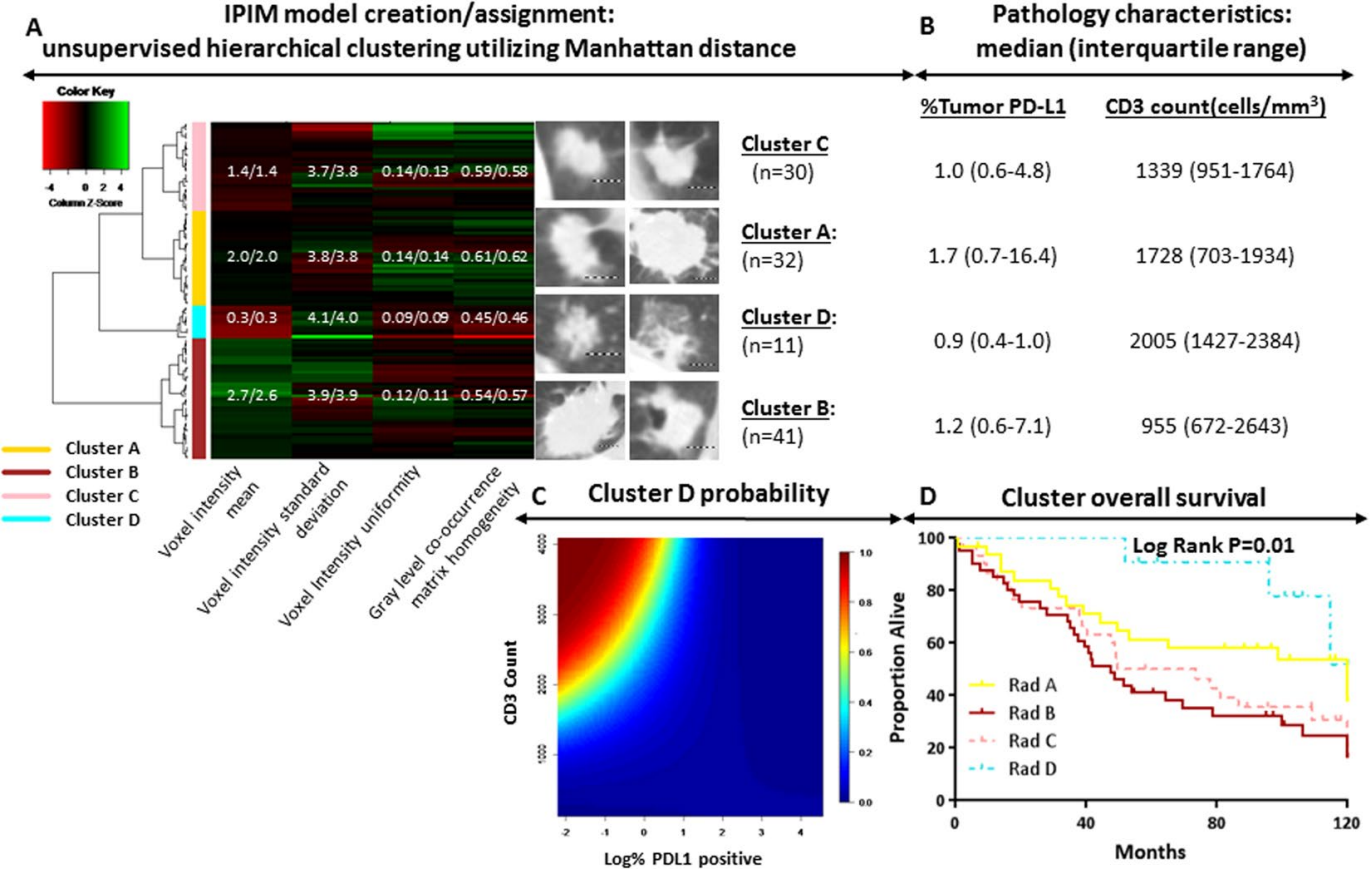
Immune-pathology informed radiomics model creation and representative radiology images (dotted bar = 1 cm) in the training cohort; green and red represent high and low value based on feature Z-score, respectively. The mean/median feature values are displayed for each radiomics cluster within the heatmap (**A**). IPIM cluster pathology characteristics shown as median (1^st^ and 3^rd^ quartiles) percent tumor PD-L1 expression and CD3 count (**B**). Probability of a patient being assigned to cluster D assignment given specified CD3 count and log (%PDL1 positivity). High and low probability designated by red and blue, respectively (**C**). Overall survival stratified by IPIM model assignment (**D**).

The IPIM consists of 4 features, 3 of which are derived by first-order analysis of image-feature intensity: mean, standard deviation, and uniformity (Fig. 3). The fourth feature is a second-order analysis of the homogeneity of the derived gray level co-occurrence matrix. IPIM features were organized into 4 clusters, named A through D. A web application was created to assign an IPIM designation for any image when the value of these 4 features was input to the application (URL: https:/biostatistics.mdanderson.org/RadiomicsSubtypingCalculator).

Multinomial regression for distinguishing the 4 IPIM clusters identified significant differences in PDL1 and CD3 expression (*P* = 0.01). These pathology characteristics are described for each cluster. Significant differences in OS were also observed among IPIM clusters (*P* = 0.01). Cluster D had a predominantly immune-activated phenotype, with high CD3 and low PDL1 expression (Fig. 3B–D), and 5-yr OS rates were correspondingly higher among patients grouped into Cluster D (91%) than for patients in Cluster A (61%), Cluster B (41%), and Cluster C (50%). This association of Cluster D with improved OS held on multivariate analysis of the training set (Table 2). The c-indices for the univariate and multivariate models were 0.61 and 0.70, respectively.

**Table 2:** Multivariate associations with survival.

**Characteristic**	**Training Set (n = 114)**			**Validation Set (n = 176)**			**Validation Set (Stage 1 only; n = 111)**		
	HR	95% CI	*P* Value	HR	95% CI	*P* Value	HR	95% CI	*P* Value
Lesion size (per cm)	1.19	1.04–1.36	0.01	1.06	0.92–1.21	0.43	1.13	0.76–1.69	0.54
N1/N2 (vs. N0)	1.36	0.77–2.40	0.30	3.75	2.07–6.81	<0.01	—	—	—
Adjuvant therapy (vs. none)	0.77	0.43–1.36	0.37	0.63	0.34–1.19	0.16	0.47	0.06–3.99	0.49
Age at surgery (per year)	1.04	1.01–1.07	0.02	1.02	0.99–1.05	0.13	1.06	1.01–1.12	0.03
Squamous histology	0.93	0.56–1.56	0.79	1.70	1.01–2.88	0.047	1.68	0.79–3.57	0.18
Current smoker (vs. former and never smoker)	1.37	0.80–2.30	0.26	1.26	0.75–2.10	0.38	0.78	0.34–1.81	0.57
Radiomics cluster (REF: Cluster D)									
Cluster A	1.52	0.42–5.46	0.52	1.44	0.68–3.08	0.34	2.91	1.15–7.36	0.02
Cluster B	3.45	1.01–11.78	0.048	1.54	0.73–3.24	0.25	1.14	0.32–4.04	0.84
Cluster C	2.81	0.80–9.86	0.11	2.75	1.24–6.10	0.01	5.30	2.08–13.50	<0.01

Abbreviations: 95% CI, 95% confidence interval; HR, hazard ratio.

### Validating the radiomics model

**Figure 4:**
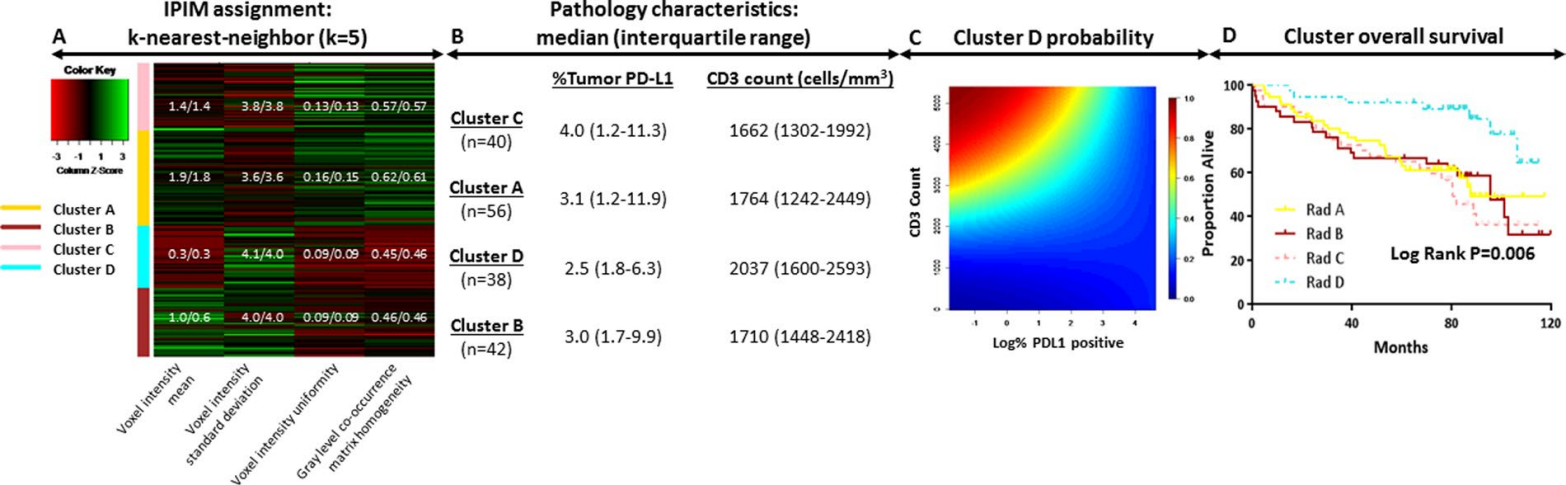
Immune-pathology informed radiomics model assignment in the validation cohort; green and red represent high and low value based on feature Z-score, respectively. The mean/median feature values are displayed for each radiomics cluster within the heatmap (**A**). IPIM cluster pathology characteristics shown as median (1^st^ and 3^rd^ quartiles) percent tumor PD-L1 expression and CD3 count (**B**). Probability of a patient being assigned to cluster D assignment given specified CD3 count and log (%PDL1 positivity). High and low probability designated by red and blue, respectively (**C**). Overall survival stratified by IPIM model assignment (**D**).

As was true in the training cluster, significant differences were noted between CD3 and PDL1 expression according to IPIM cluster assignment (*P* < 0.001). Again, pathology features of patients in Cluster D were most consistent with an immune-activated phenotype, with the lowest proportion of cells expressing PDL1 and the highest frequency of CD3+ cells (Fig. 4B,C). Survival differed significantly among the IPIM clusters (*P* = 0.006), with patients in Cluster D once again having better 5-yr OS rates (86%) than those in Cluster A (55%), Cluster B (72%), and Cluster C (75%). In the validation set the association of Cluster D with better OS held on multivariate analysis (Table 2). The c-indices for the univariate and multivariate models were 0.61 and 0.72, respectively.

We repeated the multivariate analysis considering only patients with stage I (T1-2N0M0) disease from the validation dataset (n = 111). That analysis revealed a significant association between IPIM Cluster D and improved OS (Table 2). Considering only patients with stage I disease, the c-index for the univariate and multivariate models were 0.66 and 0.73, respectively. Furthermore, we conducted sensitivity analyses with the previously described multivariate Cox model removing patients with close (<2 mm) or positive surgical margins (n = 7 and n = 9 in the training and validation cohorts, respectively). This sensitivity analysis maintained significant associations between radiomics cluster and survival (Supplemental Table S2).

## Discussion

We present a novel approach for developing a predictor of OS in patients with NSCLC based on a pathology-informed radiomics model. Past approaches produced radiomics-based stratification by association with patient outcomes (e.g., OS or progression-free survival) predominantly by using supervised analytical methods. The IPIM model presented here was identified among a selected subset of radiomics models that had been screened for associations with immune-pathology features and OS. We used two immune-pathology features to describe the immune microenvironment, CD3+ cell density and percent tumor cell PDL1 expression, to separate patients into 4 groups. This paradigm has been used previously for patients with melanoma^25^. The identified pathology groupings and patient survival were then used to create the presented IPIM model utilizing patients from the training cohort. The association of the IPIM model with patient survival and pathology were then validated in a separate cohort (the validation set). Given the immune-inhibitory function of PDL1 and the immune-activated state portended by increasing tumor infiltration by CD3+ cells, it is reasonable to assume that tumors with high CD3 infiltration and low PDL1 expression, which portend an immune-activated state, would be linked with the best patient outcomes (Fig. 1). Radiomics Cluster D was enriched for this phenotype in our analysis and correspondingly also exhibited prolonged survival (Figs 3–4).

Current reports of the use of radiomics for lung cancer have focused on 3 areas: describing patient outcomes^14–16^, discerning malignant versus benign lung nodules^26,27^, and, more recently, assessing NSCLC mutation status^18,19,28^. Efforts correlating biological phenomena with radiologic features in NSCLC have otherwise been generally limited. To our knowledge, the current study represents the first radiomics analysis to link the tumor immune microenvironment with survival. Notably, patients in this study had been treated in an era when immunotherapy was not used for NSCLC. Thus the observed differences in outcome were probably derived from the biological phenotype. We hypothesize that the differences in outcome noted in the IPIM subgroups will become more pronounced in the current era, given the increasing use of immunotherapy for this disease.

The predominant biomarker to determine eligibility for PD1 inhibitors use is tumor PDL1 expression. However, pathology analysis alone is limited by the heterogeneous expression pattern of PD-L1 on tumor cells, which varies significantly due to changes in the utilized assay, IHC antibody, and specimen size^29–31^. It is therefore plausible that the IPIM classification, in conjunction with pathology, could be used to predict or monitor response to immunotherapy. Analysis investigating the predictive power of the IPIM classification in patients treated with checkpoint inhibitors is ongoing.

Aside from the inherent weaknesses associated with retrospective reviews, various strengths and weaknesses deserve to be specifically mentioned. This study was conducted at a single institution and used only CT scanners at that institution. To facilitate outside validation we have created a web interface (URL: https:/biostatistics.mdanderson.org/RadiomicsSubtypingCalculator) to encourage its application beyond this study and at other institutions. Further, patients varied in cancer stage and era of treatment, thus receiving a variety of adjuvant treatment regimens. To adjust for this, we conducted a sensitivity analysis in our validation dataset limiting inclusion to only stage I patients (T1-2N0M0), which reaffirmed the results from the larger cohort (Table 2). Finally, an often deserving criticism of most radiomics models is that these metrics are merely a surrogate for patient size or other factors already captured by conventional staging. To ensure that our findings are robust, we controlled for lesion size in our multivariate analysis size as well as conducted a sensitivity analysis of our validation cohort limiting to only patients with stage I disease (T1-2N0M0). All adjusted analyses yielded statistically significant evidence for independent association (Table 2). Ultimately, we consider these data hypothesis-generating and deserving of validation, preferably at outside institutions.

To summarize, we present the first radiomics model to leverage immune-pathology features generated from lung lesions that had been imaged and the imaged tissue analyzed expression of immune related markers. The resulting immune-informed radiomics model yielded subtypes associated with OS and immune-pathology status after adjustment for known clinical prognostic factors. To facilitate ease of use and encourage external validation, we have created a web application in which investigators can assign IPIM classification based on their own patients’ pretreatment CT scans (URL: https:/biostatistics.mdanderson.org/RadiomicsSubtypingCalculator). We hypothesize that this entirely noninvasive model can be used in the current era to further elucidate the prognostic utility of the local immune environment and can be enhanced to predict or monitor response to immunotherapy agents, a subject of ongoing investigation.

## Methods

### Patients

Training and validation cohorts were created from two patient datasets that were temporally separated with respect to staining, processing, and quantification. Inclusion criteria were having received definitive surgery, diagnosis of non-metastatic disease, pathologically confirmed NSCLC, and lack of induction therapy. All patients had a pretreatment staging chest CT with contrast agent at our institution as part of the standard of care and had an adequate surgical pathology specimen for IHC staining. All methods were carried out in accordance with relevant guidelines and regulations. All analyses were approved by the appropriate Institutional Review Board at MD Anderson Cancer Center, with the requirement for consent waived given the retrospective design.

### Pathology analysis

Four-micron-thick sequential histologic tumor sections were obtained from a representative formalin-fixed, paraffin-embedded tumor block and used for IHC analysis. IHC staining was done using an automated staining system (BOND-MAX; Leica Biosystems, Nussloch, GmbH) with antibodies against PDL1 (clone E1L3N, dilution 1:100; Cell Signaling Technology, Beverly, MA, USA) and CD3 (T-cell lymphocytes; dilution 1:100; Dako, Carpinteria, CA, USA). Marker expression was detected with a Novocastra Bond Polymer Refine Detection kit (Leica Biosystems) with a diaminobenzidine reaction to detect antibody labeling and hematoxylin counterstaining. The slides were then scanned in an Aperio AT2 scanner (Leica Biosystems). CD3+ cells were counted by a pathologist using Aperio Image Toolbox analysis software (Aperio, Leica Biosystems) and expressed as cell density (CD3+ cells/mm^2^ of analyzed tissue). Percentage of tumor cells staining for PDL1 was quantified with the GENIE histology pattern recognition algorithm (Aperio) for automated identification of the tumor regions, under a pathologist’s supervision, via a membrane algorithm. Further details regarding staining technique are provided in the following references^32,33^.

Patients were grouped based on PDL1 staining in tumor cells and characteristics of tumor-infiltrating lymphocytes (TILs) into 4 previously established pathology groups based on T-cell infiltration and tumor PDL1 expression as follows: PD-L1^hi^CD3^hi^, PD-L1^lo^TIL^lo^, PD-L1^hi^TIL^lo^, PD-L1^lo^TIL^hi^ (Fig. 1)^25^. Optimal immune subtypes were identified from the training set with thresholds of PDL1 and CD3 expression yielding maximum association with OS as characterized by the likelihood-ratio test based on the Cox proportional hazards model. Cutpoints identified in the training cohort were then applied to the validation cohort.

### Radiomics feature extraction and characterization

Pretreatment chest CT scans with contrast agent were obtained at our institution and contoured by using 3D Slicer^34,35^. Radiomics features were extracted from contoured tumors by using IBEX (example contours displayed in Supplemental Fig. S2)^36^. We initially investigated the full set of 490 available parameters available via IBEX. Notably, radiomics analyses produce a large number of highly redundant feature sets, which necessitates variable selection to produce a sparse subset. This analytical approach, when applied to highly correlated features, often fails to produce reproducible findings across studies. Moreover, radiomics parameters are sensitive to filtering and preprocessing techniques. To overcome these limitations, we evaluated prognostic signatures on the basis of 12 features previously studied in NSCLC. These features are based on calculations from the voxel intensity: mean, kurtosis, skewness, entropy, standard deviation, and uniformity and on calculations from the gray level co-occurrence matrix: contrast, dissimilarity, energy, entropy, homogeneity, and modified homogeneity. Mathematic definitions for each feature along with the utilized filtering and preprocessing methodlogy are provided in Supplementary Tables S3–4 Features were assessed for their inter-user reproducibility by having 3 physicians use two different programs (3D Slicer and MIM Vista^34,35,38,39^) to contour a random set of 10 patients. Features exhibiting <3% deviation attributable to inter-user heterogeneity in both programs were considered “highly reproducible” and thereby given preference for radiomics model selection. The resultant Bland-Altman plots are reported in Supplementary Fig. S1.

### Cluster generation, validation, and statistical analysis

All statistical analyses were conducted with R statistical software (version 3.2.3; R Development Core Team; 2015). The ConsensusClusterPlus package was utilized to identify radiomics clusters. In addition we utilized the nnet, gplots, survival, and ConsensusClusterPlus packages. To identify robust, reproducible signatures, we avoided supervised clustering based on patient outcomes. Inter-patient dissimilarity was computed by Manhattan distance, which measures the absolute distance between two vectors. Hierarchical cluster analysis was implemented based on Ward’s method with ranks 2–6, follow Z-transformation of all radiomics parameters. Candidate clustering schemes were then evaluated for association with individual immune-pathology markers based on Spearman’s rank correlation coefficient and tested for evidence of association with the resultant immune-pathology subtypes of PDL1^hi^CD3^hi^, PDL1^lo^TIL^lo^, PDL1^hi^TIL^lo^, PDL1^lo^TIL^hi^ with likelihood-ratio tests obtained from log-linear multinomial regression^25^. Regression models estimated the relative odds of each cluster assignment given the observed expression levels of PDL1 and CD3. Six candidate signatures were selected as highly associated with immune pathology based on the training set. Thereafter, clusters from the selected feature sets were evaluated for association with OS in univariate analysis based on the log-rank test. To ensure that the inferred relationships represented independent lines of association from standard clinical knowledge, Cox proportional hazards regression was applied to adjust the radiomics estimators for the partials of effects of prognostic clinical factors and treatment factors: lesion size, N-status, histologic subtype, age at surgery, and whether adjuvant therapy was administered.

Model validation was conducted in an independent cohort. Similar to the pathology model, cutpoints identified in the training cohort were then applied to the validation cohort. For each of the 6 selected signatures, radiomics cluster assignments were obtained independently for each patient in the validation set by evaluating their extent of pairwise similarity with each patient in the training set. Each validation patient was assigned to one training cluster based on the k-nearest-neighbor algorithm applied to similarity measures obtained from consensus clustering using k = 5^40^. Consensus clustering was implemented based on the hierarchical clustering algorithm with Manhattan distance function using rank size fixed at the number of clusters-levels for the considered signature. All tests were two-sided where applicable and considered significant when *P* ≤ 0.05.

## Electronic supplementary material

Supplementary Information
