# Supplementary material for: Development of an Immune-Pathology Informed Radiomics Model for Non-Small Cell Lung Cancer
Source: Sci Rep. 2018 Jan 31;8:1922. doi: 10.1038/s41598-018-20471-5 (PMC5792427; doi:10.1038/s41598-018-20471-5)
Supplement: Supplementary file 1 — Supplementary Information [file 41598_2018_20471_MOESM1_ESM.pdf]

Development of an Immune-Pathology Informed Radiomics Model for Non-Small Cell Lung Cancer

Supplemental Items

Authors:
Chad Tang
Brian Hobbs
Ahmed Amer
Xiao Li
Carmen Behrens
Jaime Rodriguez Canales
Edwin Parra Cuentas
Pamela Villalobos
David Fried
Joe Y. Chang
David S. Hong
James W. Welsh
Boris Sepesi
Laurence Court
Ignacio I. Wistuba
Eugene J. Koay

		SLICER	MIMvista
Voxel intensity metrics	Mean*	± 1.3%	± 1.5%
	Kurtosis	± 10.2%	± 7.4%
	Skewness	± 12.5%	± 10.6%
	Entropy	± 0.7%	± 0.9%
	Std deviation*	± 0.3%	± 0.4%
	Uniformity*	± 1.6%	± 2.4%
Gray level co-occurrence	Contrast	± 3.1%	± 3.1%
	Dissimilarity	± 5.4%	± 5.2%
	Energy	± 5.1%	± 6.2%
	Entropy	± 1.5%	± 1.7%
	Homogeneity	± 1.1%	± 0.6%
	Modified Homogeneity	± 1.6%	± 0.6%

*indicates features utilized in the immune pathology informed model (IPIM)

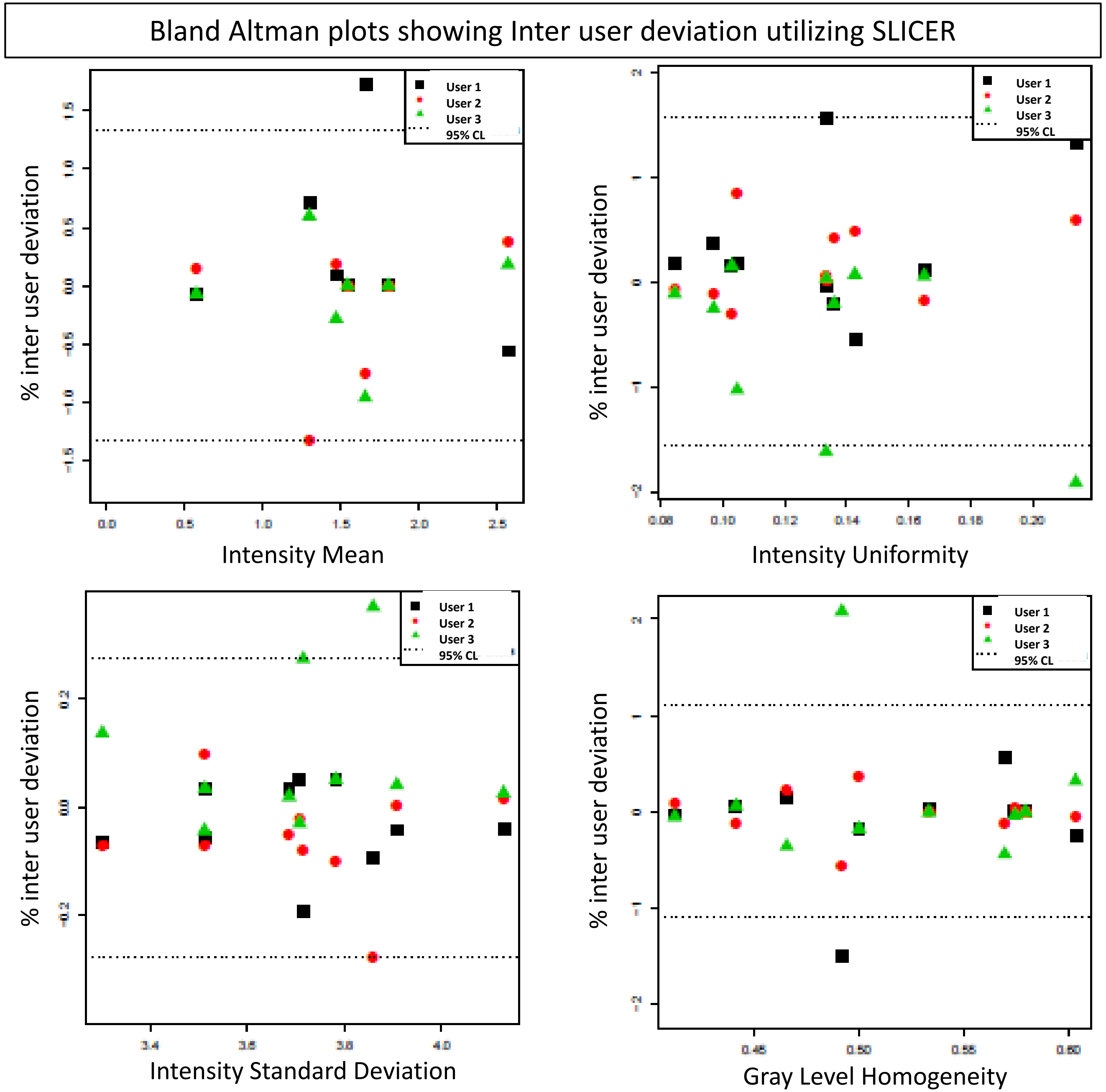

Supplementary Fig. S1. Inter-user deviation comparing radiomics features created from contours from 3 different radiation oncologists in 2 different contouring programs (SLICER and MIMvista). Representative Bland Altman plots are shown for 4 highly reproducible features (defined as

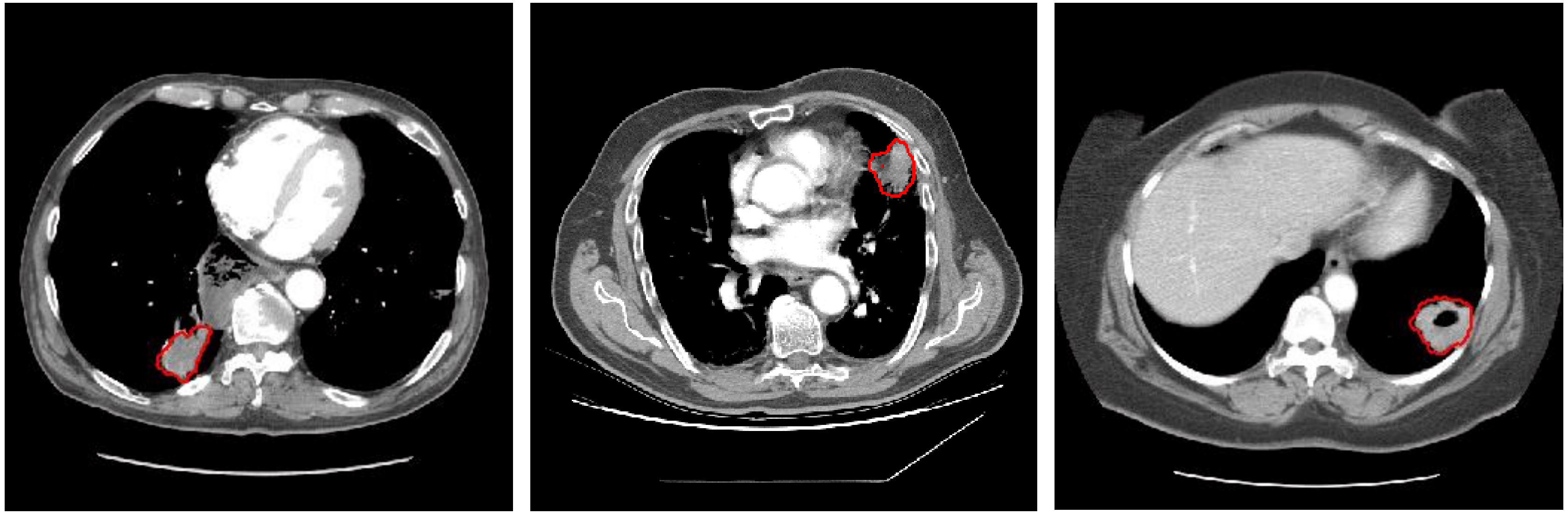

Supplementary Fig. S2. Representative segmentations of lung tumors.

Supplemental Table S1. Multivariate associations with survival assessing pathology status

	Training Set (n=114)			Validation Set (n=176)		
**Characteristic**	HR	95% CI	*P* Value	HR	95% CI	*P* Value
Lesion size (per cm)	1.19	1.03-1.36	0.02	1.04	0.92-1.18	0.53
N1/N2 (vs. N0)	1.16	0.67-2.02	0.60	4.92	2.70-8.96	<0.01
Adjuvant therapy (vs. none)	0.84	0.47-1.52	0.57	0.58	0.30-1.12	0.10
Age at surgery (per year)	1.04	1.01-1.07	0.02	1.03	1.00-1.07	0.04
Squamous histology	1.03	0.60-1.77	0.91	1.50	0.92-2.45	0.11
Current smoker (vs. former and never smoker)	1.14	0.67-1.95	0.62	1.24	0.74-2.09	0.41
Radiomics cluster (REF: $CD3^{lo}PDL1^{hi}$)						
$CD3^{hi}PDL1^{hi}$	1.06	0.44-2.56	0.89	0.37	0.20-0.69	<0.01
$CD3^{hi}PDL1^{lo}$	0.31	0.10-0.95	0.04	0.14	0.03-0.57	<0.01
$CD3^{lo}PDL1^{lo}$	0.87	0.50-1.52	0.62	1.24	0.74-2.09	0.01

Abbreviations: 95% CI, 95% confidence interval; HR, hazard ratio
Histology groupings are as follows, $CD3^{lo}PDL1^{hi}$: CD3 low and PDL1 high, $CD3^{hi}PDL1^{hi}$: CD3 high and PDL1 high, $CD3^{hi}PDL1^{lo}$: CD3 high and PDL1 low, $CD3^{lo}PDL1^{lo}$: CD3 low and PDL1 low.

Supplemental Table S2. Sensitivity analysis, multivariate associations with survival in patients with negative resection margins

	**Training Set (n=107)**			**Validation Set (n=167)**			**Validation Set (Stage 1 only; n=107)**		
**Characteristic**	HR	95% CI	*P* Value	HR	95% CI	*P* Value	HR	95% CI	*P* Value
Lesion size (per cm)	1.18	1.02-1.36	0.03	0.96	0.81-1.12	0.59	1.11	0.73-1.69	0.62
N1/N2 (vs. N0)	1.61	0.86-3.01	0.30	3.92	2.09-7.36	<0.01	—	—	—
Adjuvant therapy (vs. none)	0.64	0.34-1.19	0.16	0.76	0.40-1.47	0.42	0.55	0.06-4.68	0.58
Age at surgery (per year)	1.05	1.01-1.08	0.02	1.01	0.98-1.04	0.44	1.06	1.00-1.12	0.04
Squamous histology	0.81	0.47-1.41	0.81	1.70	1.01-2.88	0.047	1.75	0.80-3.87	0.16
Current smoker (vs. former and never smoker)	1.26	0.71-2.24	0.43	1.06	0.61-1.83	0.84	0.76	0.31-1.82	0.57
Radiomics cluster (REF: Cluster D)									
Cluster A	1.59	0.44-5.81	0.48	1.58	0.71-3.52	0.26	2.77	1.07-7.20	0.04
Cluster B	3.71	1.07-12.80	0.04	1.52	0.69-3.38	0.30	1.00	0.23-4.33	>0.99
Cluster C	2.79	0.78-9.92	0.11	2.80	1.22-6.43	0.02	4.94	1.88-13.00	<0.01

Abbreviations: 95% CI, 95% confidence interval; HR, hazard ratio

Supplemental Table S3: Voxel intensity metrics

Feature	Feature Algorithm	Ref	Preprocessing	Preprocessing Parameters
Mean	$\sum_{i=1}^{Ng} i * h(i)$	3,4,5	Threshold_Image_Mask<br>Log_Filter	ThresholdLow=900; ThresholdHigh=8000; ErosionDist=0;<br>Size=15; Sigma=1; FillROIOutOn=1; FillROIOutValue=5000;
Kurtosis	$\frac{\sum_{i=1}^{Ng}(i-\sum_{i=1}^{Ng} i * h(i))^4 * h(i)}{(\sum_{i=1}^{Ng}(i-\sum_{i=1}^{Ng} i * h(i))^2 * h(i))^2}$	3,4,5	Threshold_Image_Mask	ThresholdLow=900; ThresholdHigh=8000; ErosionDist=0;
Skewness	$\frac{\sum_{i=1}^{Ng}(i-\sum_{i=1}^{Ng} i * h(i))^3 * h(i)}{(\sum_{i=1}^{Ng}(i-\sum_{i=1}^{Ng} i * h(i))^2 * h(i))^{3/2}}$	3,4,5	BitDepthRescale_Range	RangeMin=0; RangeMax=4096; RangeFix=1; BitDepth=8;
Entropy	$-\sum_{i=1}^{Ng} h(i) * \log(h(i))$	3,4,5	Threshold_Image_Mask	ThresholdLow=900; ThresholdHigh=8000; ErosionDist=0;
Standard Deviation	$\sqrt{\sum_{i=1}^{Ng}(i-\sum_{i=1}^{Ng} i * h(i))^2 * h(i)}$	3,4,5		
Uniformity	$\sum_{i=1}^{Ng} h(i)^2$	4,5		

Ng is the number of grey levels, h(i) is the probability of intensity i. All labels and parameter nomenclature are in reference to that utilized in IBEX.

Supplemental Table S4: Gray level co-occurrence metrics

Feature	Feature Algorithm	Ref	Preprocessing	Preprocessing Parameters
Contrast	$\sum_{n=0}^{N_g-1} n^2 \left\{\sum_{i=1}^{Ng} \sum_{j=1}^{Ng} p(i,j) \middle\| \|i-j\| = n\right\}$	1,2	Threshold_Image_Mask	ThresholdLow=900; ThresholdHigh=8000; ErosionDist=0;
Dissimilarity	$\sum_{i=1}^{Ng}\sum_{j=1}^{Ng} \|i-j\| * p(i,j)$	1,2	BitDepthRescale_Range	RangeMin=0; RangeMax=4096; RangeFix=1; BitDepth=8;
Energy	$\sum_{i=1}^{Ng}\sum_{j=1}^{Ng} p(i,j)^2$	1,2		
Entropy	$-\sum_{i=1}^{Ng}\sum_{j=1}^{Ng} p(i,j) * \log(p(i,j))$	1,2		
Homogeneity	$\sum_{i=1}^{Ng}\sum_{j=1}^{Ng} \frac{1}{1+\|i-j\|} * p(i,j)$	1,2		
Modified Homogeneity	$\sum_{i=1}^{Ng}\sum_{j=1}^{Ng} \frac{1}{1+(i-j)^2} * p(i,j)$	2		

Ng is the number of grey levels, p(i,j) is the (i,j)th element in the Grey Level Cooccurrence Matrix (GLCM) respectively. All labels and parameter nomenclature are in reference to that utilized in IBEX.

References:

1. L. Soh and C. Tsatsoulis. Texture analysis of sar sea ice imagery using gray level co-occurances matrices. IEEE Trans. on Geoscience and Remote Sensing, 37(2):780-795, 1999.
2. Hugo J. W, Sara Cavalho, et al. Decoding tumour phenotype by noninvasive imaging using a quantitative radiomics approach. Nat. Commun. 2014; 5: 4006.
3. Basu S. Developing Predictive Models for Lung Tumor Analysis. University of South Florida; 2012.
4. Cunliffe AR, Armato SG, 3rd, Fei XM, et al. Lung texture in serial thoracic CT scans: registration-based methods to compare anatomically matched regions. Med Phys. 2013;40(6):061906.
5. Fried DV, Tucker, S. L., Zhou, S., Liao, Z., Mawlawi, O., Ibbott, G., Court, L. E. . Prognostic value and reproducibility of pretreatment CT texture features in stage III Non-Small-Cell lung cancer Int J Radiation Oncol Biol Phys. 2014.
